# Multipathway modulation of neuroinflammation and oxidative stress by hesperidin in a rat single prolonged stress model of post‐traumatic stress disorder

**DOI:** 10.1002/ame2.70244

**Published:** 2026-08-06

**Authors:** Godson Emeka Anyanwu, Ogan Christopher Akanaku, Augustine Oviosun, Precious E. Ekwueme, Paul Anyiom Odey, Chima Paul Okechukwu Ugwu, Chinyere Nkemjika Anyanwu

**Affiliations:** ^1^ Department of Anatomy, Faculty of Biomedical Sciences Kampala International University Kampala Uganda; ^2^ Department of Anatomy, Faculty of Basic Medical Sciences University of Cross River State Calabar Nigeria; ^3^ Department of Anatomy, Faculty of Basic Medical Sciences, College of Medicine University of Nigeria, Enugu Campus Enugu Nigeria; ^4^ Department of Anatomy, Faculty of Basic Medical Sciences University of Calabar Calabar Cross River State Nigeria; ^5^ Directorate of Research, Innovation, Consultancy and Extension (RICE) Kampala International University Kampala Uganda; ^6^ Department of Microbiology and Immunology, Faculty of Biomedical Sciences Kampala International University Kampala Uganda

**Keywords:** amygdala, hesperidin, hippocampus, neuroinflammation, oxidative stress, post‐traumatic stress disorder, prefrontal cortex, single prolonged stress

## Abstract

**Background:**

Post‐traumatic stress disorder (PTSD) is a complicated neuropsychiatric disorder that is marked by long‐term neuroinflammation, oxidative stress, and poor neuroplasticity in stress‐sensitive brain areas. Hesperidin is a citrus‐derived flavanone glycoside that has been reported to have multitarget neuroprotective effects with antioxidant, anti‐inflammatory, and neurotrophic regulatory activities. However, its integrated effects across convergent pathological pathways in validated PTSD models remain insufficiently defined.

**Methods:**

Male Wistar rats were subjected to the single prolonged stress (SPS) paradigm and treated with hesperidin (50, 100, and 200 mg/kg), fluoxetine (20 mg/kg), or vehicle for 14 days. Biochemical assessments (malondialdehyde [MDA], catalase [CAT], superoxide dismutase [SOD]) and immunohistochemical measurements of neuronal and glial integrity (NeuN, glial fibrillary acidic protein [GFAP], brain‐derived neurotrophic factor [BDNF]) in the hippocampus, amygdala, and prefrontal cortex were performed.

**Results:**

SPS exposure induced marked oxidative imbalance, evidenced by increased MDA levels and reduced CAT activity, alongside significant upregulation of tumor necrosis factor α (TNF‐α), interleukin 6 (IL‐6), and IL‐1β, increased astrocytic reactivity (GFAP), and reduced BDNF expression. Hesperidin treatment significantly attenuated lipid peroxidation, partially restored CAT activity, and suppressed pro‐inflammatory cytokines, with the most pronounced effect observed for IL‐1β. Notably, hesperidin reduced astrocytic activation and preserved neuronal morphology, while enhancing BDNF expression in a dose‐dependent manner, with optimal effects at 50–100 mg/kg. Conversely, there was little group variation in the activity of SOD, implying pathway‐selective redox regulation.

**Conclusion:**

Hesperidin has multipathway, coordinated, modulatory effects on oxidative stress, neuroinflammation, glial activation, and neurotrophic signaling in the SPS model of PTSD. These findings support its utility as a mechanistic probe for interrogating convergent stress‐related neuropathological pathways.

## Graphical abstract

Single prolonged stress (SPS) induces post‐traumatic stress disorder (PTSD)‐like neuropathology characterized by oxidative stress, neuroinflammation, astrocytic reactivity, neuronal damage, and reduced neurotrophic support in the hippocampus, amygdala, and prefrontal cortex. Hesperidin counteracts these effects by attenuating lipid peroxidation and pro‐inflammatory cytokines, restoring antioxidant defenses, and preserving neuronal integrity. Overall, hesperidin has multipathway neuroprotective effects in experimental rat model of PTSD.

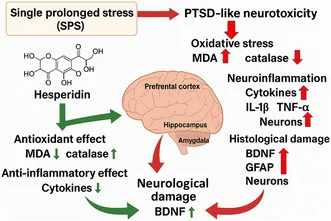

## 1 INTRODUCTION

Post‐traumatic stress disorder (PTSD) is a long‐term neuropsychiatric disorder characterized by maladaptive fear processing and affective and cognitive impairment that results after exposure to trauma. Though historically viewed as a purely psychological disorder, there is a growing representative clinical and preclinical body of evidence indicating that PTSD is a disease with an underlying neurobiological pathology of persistent neurobiological dysregulation.^1^, ^2^ Such changes encompass sustained oxidative disequilibrium, continuous neuroinflammatory response, and poor neuroplasticity in the stress‐sensitive brain pathways of the hippocampus, amygdala, and prefrontal cortex.^3^, ^4^

Among the available experimental paradigms, the single prolonged stress (SPS) model is one of the most extensively validated rodent models of post‐traumatic stress disorder (PTSD).^4^, ^5^ SPS is a reliable model for reproducing key neuroendocrine features of the disorder, such as hypothalamic–pituitary–adrenal (HPA) axis dysregulation, fear extinction impairment, increased anxiety‐like behavior and long‐lasting molecular and structural brain changes.^6^, ^7^ Notably, SPS provokes chronic oxidative stress and neuroinflammatory events in corticolimbic areas, which offer a solid platform to challenge experimental treatments that act on convergent pathological pathways rather than single neurotransmitter systems.^8^, ^9^, ^10^

The growing body of evidence suggests that neuroinflammation and oxidative stress can be the primary mechanisms of PTSD chronicity rather than an epiphenomenal consequence.^11^, ^12^ Sustained elevations of pro‐inflammatory cytokines, such as interleukin 1β (IL‐1β), IL‐6, and tumor necrosis factor α (TNF‐α), increased lipid peroxidation products, and decreased antioxidant enzyme activity have consistently been observed in PTSD patients and rodents exposed to SPS.^13^, ^14^ Those molecular disruptions are intertwined with astrocytic reactivity, neuronal susceptibility, and lowering of brain‐derived neurotrophic factor (BDNF) that are all related to impeded synaptic plasticity, structural fragility, and behavioral rigidity.^15^, ^16^, ^17^, ^18^

Existing first‐line pharmacotherapy of PTSD, especially selective serotonin reuptake inhibitors (SSRIs), has a small effect on symptoms and few results on reversing PTSD‐related oxidative injury, neuroinflammatory stimulation, or structural brain modifications.^19^, ^20^ These limitations underscore the need for experimental strategies that interrogate multipathway modulation within validated disease models rather than approaches narrowly focused on monoaminergic signaling.

Hesperidin, a citrus‐derived flavanone glycoside, has been widely employed as a biological probe in experimental neuroscience to investigate redox regulation, inflammatory signaling, and neurotrophic support.^21^, ^22^, ^23^, ^24^ Hesperidin has been demonstrated to mediate the antioxidant enzyme response, anti‐inflammatory cytokine response, and plastics activity related to BDNF in models of central nervous system injury and stress across different models.^25^, ^26^, ^27^ Although this now accumulated evidence indicates a role of hesperidin in the integrated oxidative‐inflammatory‐neurotrophic cascade of the PTSD models that have been proven to be validated, the impact of hesperidin on the integrated oxidative‐inflammatory‐neurotrophic axis across the multiple stress‐sensitive brain regions has not been clearly defined.

Recent evidence indicates that plant‐derived flavonoids such as hesperidin and others exert pleiotropic neuroprotective effects through coordinated modulation of oxidative stress, neuroinflammatory signaling, and neurotrophic pathways.^12^, ^28^, ^29^ These compounds have been shown to attenuate HPA axis dysregulation, suppress pro‐inflammatory cytokine cascades, and enhance BDNF signaling in experimental models of neuropsychiatric disorders. Such multitarget mechanisms are increasingly recognized as critical in conditions like PTSD, where convergent network dysfunction, rather than isolated neurotransmitter imbalance, underlies disease progression.

To enhance translational relevance of experimental studies, behavioral validation of PTSD‐like phenotypes is required. A more recent study with the same SPS paradigm and dosing regimen has shown that SPS causes strong anxiety‐ and depression‐like behaviors, impaired exploratory behavior, and anhedonia, as measured by the elevated plus maze, open‐field test, and sucrose preference test, respectively.^30^ Hesperidin, in that study, alleviated behavioral impairments, which offer phenotypic background to further mechanistic studies of stress‐induced neuropathology.

Based on this proven behavior framework, the current study was set up to mechanically question the hypothesis that hesperidin regulates the action of SPS‐induced oxidative stress, neuroinflammatory signaling, astrocytic reactivity, and neuronal integrity in the main PTSD‐relevant brain areas. We hypothesized that hesperidin would (i) attenuate oxidative damage, (ii) suppress pro‐inflammatory cytokine expression, (iii) reduce reactive astrogliosis, and (iv) preserve neuronal phenotype and neurotrophic signaling, with dose‐dependent effects. Fluoxetine served as a reference comparator to put the specificity of pathway in perspective instead of therapeutic superiority.

## 2 MATERIALS AND METHODS

### 2.1 Ethical approval and reporting standards

All the experiments were conducted according to the ARRIVE 2.0 recommendations and the National Institutes of Health Guide on the Care and Use of Laboratory Animals. Ethical approval was obtained from the Research Ethics Committee of the College of Medicine, University of Nigeria, Enugu Campus (approval no.: COMHREC/2025/01/002).

### 2.2 Animals and housing

Wistar rats (48 adult males; 160–200 g; 80–120 days old) were procured in the institutional animal facility. Animals were housed under controlled environmental conditions (22 ± 2°C; 12‐h light/dark cycle; 50%–60% relative humidity) with ad libitum access to standard chow and water. According to the SPS protocol, rats were group‐housed in the acclimatization phase and singly housed in the post‐SPS isolation phase.

### 2.3 Power analysis and sample size estimation

The a priori determination of the sample size was done using a single prespecified primary contrast (SPS + hesperidin 200 mg/kg vs. SPS‐only) and a primary biochemical endpoint (e.g., hippocampal TNF‐α or malondialdehyde [MDA]). Using *α* = 0.05 (two‐sided) with 80% power (*β* = 0.20), the past literature of SPS and internal pilot assay accuracy suggested an estimated within‐group coefficient of variation of 20% and a small biologically significant effect of a 30% reduction compared to SPS. This was equivalent to a standardized effect size (Cohen's *d*) of 1.5.

Using a two‐sample *t*‐test approximation for equal group sizes:

$$n\;\mathrm{per}\;\text{group}=\left[2\times {\left(z_{₁}-\alpha /₂+z_{₁}-\beta \right)}^{2}\right]/d^{2}.$$

This resulted in a minimum of seven animals in a group. To take the potential attrition or tissue loss into account, group sizes were adjusted by about 10%–15%, yielding a final sample size of eight rats per group in six experimental groups. The main inference was on the comparison of hesperidin 200 mg/kg versus SPS‐only, and the others were viewed as exploratory.

### 2.4 Experimental design, randomization, and blinding

Animals were randomly assigned to six experimental groups (*n* = 8 per group): control, SPS‐only, SPS + fluoxetine (20 mg/kg), and SPS + hesperidin (50, 100, or 200 mg/kg). Randomization was performed using a computer‐generated random number sequence prepared by a researcher not involved in behavioral scoring, tissue processing, or outcome quantification. Group assignments were concealed using coded identifiers. Treatment solutions were labeled with nondescriptive codes, and investigators responsible for behavioral assessment, biochemical assays, histology, imaging, and image quantification remained blinded to group allocation throughout data collection and preliminary analysis. Group codes were only broken after the completion of outcome measurements and initial statistical processing.

### 2.5 PTSD induction using single prolonged stress protocol

PTSD‐like pathology was induced according to the established SPS protocol.^31^ Rats were subjected sequentially to restraint stress for 2 h, followed immediately by forced swim stress for 20 min in a cylindrical tank filled with water maintained at room temperature. After a 15‐min recovery period, animals were exposed to diethyl ether until loss of consciousness. Thereafter, animals were returned to their home cages and singly housed for seven consecutive days, consistent with the incubation period commonly used to allow the development of persistent PTSD‐like neurobiological and behavioral alterations. This SPS sequence was applied once. In this study, the SPS model applied to induce HPA axis dysregulation was well validated in the literature,^10^, ^11^, ^12^ and model validation was supported by behavioral phenotypes and convergent molecular and cellular stress markers.

### 2.6 Neurobehavioral assessment (contextual validation)

Neurobehavioral assessments were conducted in this cohort using the SPS protocol and dosing regimen to validate stress‐induced phenotypes, and the results have been reported.^30^ Behavioral validation of the SPS model was conducted using the elevated plus maze (EPM), open‐field test (OFT), and sucrose preference test (SPT), assessing anxiety‐like behavior, exploratory activity, and anhedonia, respectively. These behavioral outcomes, obtained using the same experimental cohort and protocol, have been previously reported and are referenced here to provide phenotypic context for the present mechanistic analyses; full behavioral datasets and statistical analyses are reported in the prior publication.

### 2.7 Drug preparation and administration

Hesperidin (97% purity; Sigma‐Aldrich St. Louis, MO, USA; Cat. No. H5254) and fluoxetine hydrochloride (Sigma‐Aldrich, St. Louis, MO, USA) and fluoxetine were orally dosed using gavage once daily and over 14 days straight after the SPS exposure. The dosages were chosen according to previous rodent experiments that showed that this concentration exhibited neuropharmacological activity.^32^, ^33^ Control and SPS‐only animals received equivalent volumes of vehicle (0.9% saline; 1 mL/100 g body weight). All administrations were performed at approximately the same time each day to minimize circadian variability.

### 2.8 Tissue collection and biochemical analyses

After the last day of treatment, animals were deprived of food overnight (12 h) with access to water. The next day animals were anesthetized with sodium pentobarbital (50 mg/kg, intraperitoneal) to a surgical state of anesthesia and euthanized to reduce biochemical confounding caused by hypoxia.

Entire brains were quickly excised and put on an ice‐cooled dissection table. The trained investigator used the Paxinos and Watson rat brain atlas to bilaterally remove the hippocampus, prefrontal cortex, and amygdala. Tissues were snap‐frozen in liquid nitrogen within 2 min of acquisition and stored at −80°C pending analysis.

In biochemical assays, the tissues were frozen (1:10 w/v) in ice‐cold phosphate buffer (0.1 mol/L, pH 7.4) that included a cocktail of protease inhibitors (Protease Inhibitor Cocktail Tablets, Complete™, Roche Diagnostics GmbH, Mannheim, Germany; Cat. No. 11836153001). Homogenates were centrifuged at 10 000 × *g* for 10 min at 4°C, and the supernatant was aliquoted for oxidative stress and cytokine measurements. All biochemical parameters were normalized to protein concentration determined by the Bradford assay and expressed as the units of all parameters per milligram of protein. Biochemical parameters were all adjusted to protein concentration calculated using the Bradford assay and presented as units per milligram of protein.

Biochemical analyses on prespecified numbers of animals in each group were performed according to ARRIVE 2.0 on outcome‐specific sampling; all statistical analyses took into consideration the predefined experimental unit.

### 2.9 Evaluation of oxidative stress markers

#### 2.9.1 Malondialdehyde

Lipid peroxidation was measured by the thiobarbituric acid reactive substances (TBARS) assay following the method of Buege and Aust,^34^ with slight modifications. Absorbance was determined at 532 nm using UV–Vis spectrophotometer (Shimadzu, Japan). MDA concentration was determined with an extinction coefficient of 1.56 × 105 M^−1^ × cm^−1^ and was calculated in nmol/mg protein.

#### 2.9.2 Superoxide dismutase

Superoxide dismutase (SOD) activity was measured as described by Misra and Fridovich,^35^ based on the inhibition of epinephrine autooxidation spectrophotometrically at 480 nm. One unit of SOD activity was taken as the amount of enzyme needed to inhibit 50% epinephrine autooxidation under assay conditions and was expressed as units/mg protein.

#### 2.9.3 Catalase

Catalase (CAT) activity was measured according to the method of Aebi^36^ by measuring the breakdown of hydrogen peroxide at 240 nm. Enzyme activity was expressed as unit/mg protein, where 1 unit equals activity of the enzyme decomposing 1 mmol H_2_O_2_/min at the assay condition.

All oxidative stress assays were performed in duplicate using appropriate reagent blanks and controls. Investigators performing the biochemical measurements were masked to the group to which each participant belonged.

### 2.10 Pro‐inflammatory cytokine quantification

Tissue supernatants were used to measure TNF‐α, IL‐6, and IL‐1β using commercially available rat‐specific enzyme immunoassays (EIA) (Elabscience Biotechnology, Wuhan, China; TNF‐α: E‐EL‐R0019; IL‐6: E‐EL‐R0015; IL‐1β: E‐EL‐R0012). Assays were performed following the manufacturer's instructions.

Absorbance was quantified at 450 nm wavelength using a microplate reader (BioTek Instruments, Inc., Winooski, VT, USA). Cytokine concentrations were determined from standard curves using known concentrations and normalized for total protein content. Samples were assayed in duplicate, and the intra‐ and interassay coefficients of variation were below 10%. Results are expressed as pg/mg protein.

### 2.11 Histology and immunohistochemistry

#### 2.11.1 Tissue fixation and sectioning

The samples used in histological studies were anesthetized with ketamine and perfused pericardially with 0.1 mol/L phosphate‐buffered saline (PBS), followed by neutral‐buffered formalin. Postfixation of brains was followed by paraffin embedding and coronal sectioning using a rotary microtome (Leica RM2125). The dorsal hippocampus (CA1‐CA3 and dentate gyrus), basolateral amygdala, and medial prefrontal cortex were regions of interest.

#### 2.11.2 Nissl staining

Sections were stained with 0.1% cresyl violet acetate for 15 min, differentiated with 0.25% acetic alcohol, dehydrated in graded alcohols, cleared, and covered with a slide. The light microscopic analysis was performed to determine neuronal morphology and Nissl substance integrity.

#### 2.11.3 Immunohistochemistry

Sections were put through heat‐mediated antigen retrieval (citrate buffer, pH 6.0; 95–100°C; 60 min), endogenous peroxidase quenching by 3% hydrogen peroxide and blocking with 10% normal goat serum. Overnight incubation of sections at 4°C was done using the following primary antibodies (Elabscience, Wuhan, China):

Marker antibody‐type dilution catalog no.

BDNF anti‐BDNF polyclonal 1:50 E‐AB‐11510.

GFAP anti‐GFAP polyclonal 1:300 E‐AB‐13426.

NeuN anti‐NeuN polyclonal 1:300 E‐AB‐33221.

After incubating with the right secondary antibodies, the immunoreactivity was observed with diaminobenzidine (DAB). The sections were counterstained with Harris hematoxylin, dried, and stained. Negative controls were performed simultaneously and without the inclusion of the primary antibodies.

### 2.12 Image acquisition and quantification

The photomicrographs were taken out using a Leica ICC50 camera at 100× and 400× magnification. ImageJ software (NIH, Bethesda, MD, USA) was used to measure the number of astrocytes positive to GFAP, those positive to NeuN, and those positive to BDNF‐immunoreactive cells.

To minimize sampling bias, several regions were analyzed on each animal, and three nonoverlapping fields were counted in each region using consistent cytoarchitectural landmarks. Quantitative data are averages of animals, and the animal was treated as an experimental unit. Although there could be slight differences in rostrocaudal sectioning, every area was located according to known anatomical standards as opposed to stereotaxic coordinates.

### 2.13 Statistical analysis

Statistical tests were performed using SPSS version 17.0 (SPSS Inc., Chicago, IL, USA). Behavioral measures collected over time were analyzed using two‐way repeated‐measures analysis of variance (ANOVA) (within‐subject factor: time; between‐subject factor: group), followed by post‐hoc testing where appropriate, as previously reported. One‐way ANOVA was applied to biochemical and histological endpoints. The primary inference was on the prespecified comparison between the SPS + hesperidin (200 mg/kg) and SPS‐only groups, with additional group comparisons considered exploratory. The normality and homogeneity of variance assumptions were examined, and data were log transformed if necessary. A two‐tailed *p*‐value of <0.05 was considered statistically significant.

Pearson's correlation analyses were performed to investigate the relationship between molecular markers and behavioral indices, with correlation coefficients (*r*) and *p*‐values presented. Data are presented as mean ± standard error of the mean (SEM). All analyses were performed by investigators blinded to group allocation.

## 3 RESULTS

### 3.1 SPS induces oxidative, inflammatory, and structural brain alterations consistent with PTSD‐like neurobiology

Exposure to the SPS paradigm resulted in robust and lasting neurobiological changes that were consistent with established PTSD‐like phenotypes. Relative to control animals, SPS‐exposed rats showed significant oxidative imbalance, increased expression of pro‐inflammatory cytokines, increased astrocytes reactivity, and significant neuronal loss in stress‐sensitive brain regions, including the hippocampus, basolateral amygdala, and medial prefrontal cortex.

These behavioral outcomes were assessed using elevated plus maze, open‐field, and sucrose preference paradigms, and detailed results have been reported in our earlier publication,^30^ thereby confirming the construct and face validity of the current model.

### 3.2 SPS induces significant oxidative imbalance characterized by increased lipid peroxidation and reduced catalase activity

**FIGURE 1:**
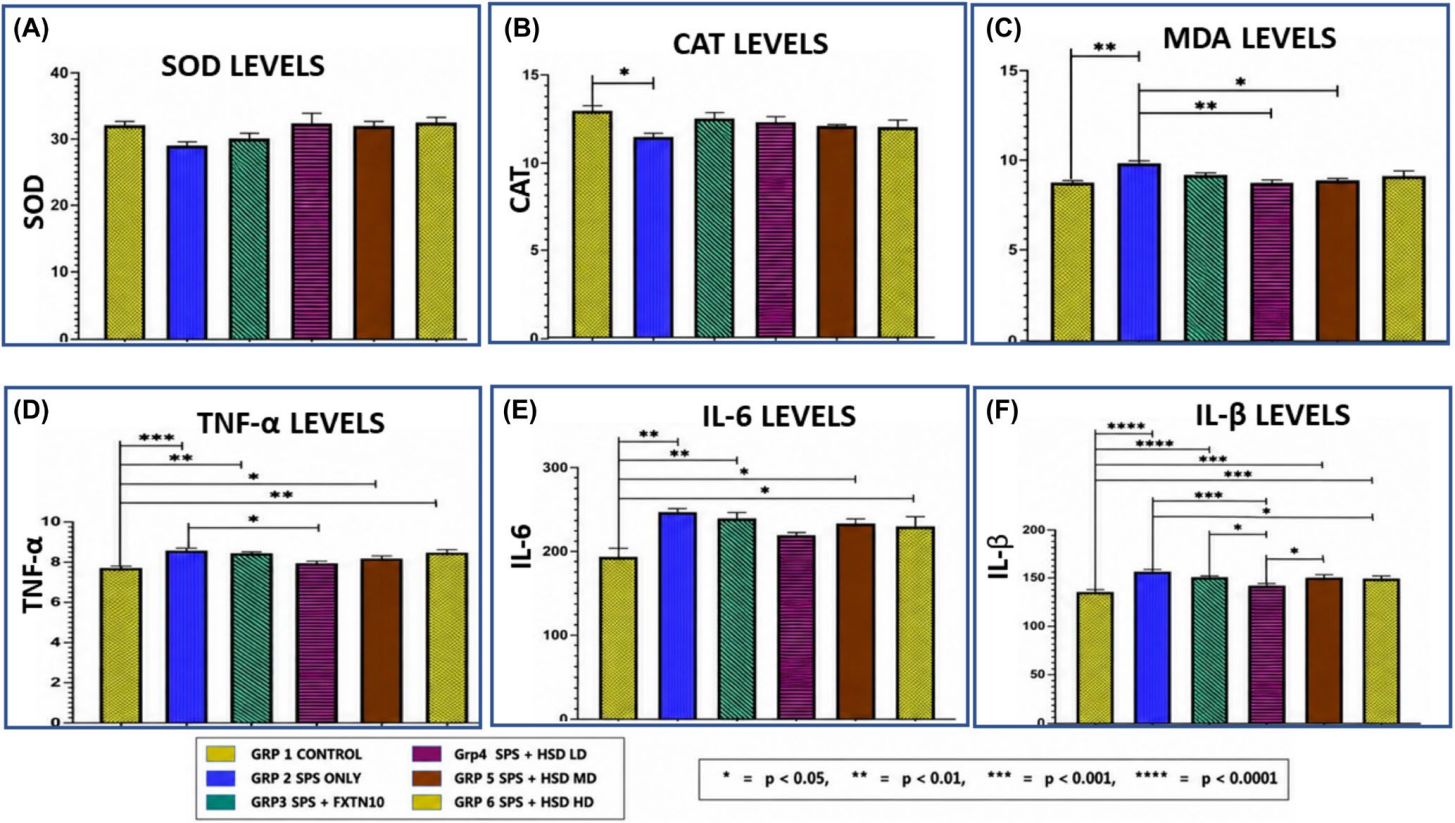
Effects of hesperidin on oxidative stress biomarkers and pro‐inflammatory cytokines in rats exposed to single prolonged stress (SPS). (A) Superoxide dismutase (SOD) activity, (B) catalase (CAT) activity, and (C) malondialdehyde (MDA) levels in brain tissue following SPS exposure and treatment with hesperidin (50, 100, and 200 mg/kg) or fluoxetine (FXT). SPS significantly increased lipid peroxidation, as evidenced by elevated MDA levels and reduced CAT activity, whereas SOD activity remained relatively unchanged across groups. Hesperidin attenuated oxidative stress by reducing MDA levels and partially restoring CAT activity. (D) Tumor necrosis factor‐α (TNF‐α), (E) interleukin‐6 (IL‐6), and (F) interleukin‐1β (IL‐1β) concentrations in brain tissue. SPS induced marked neuroinflammatory responses characterized by elevated cytokine expression. Hesperidin treatment significantly reduced TNF‐α, IL‐6, and IL‐1β levels, with the most pronounced suppression observed for IL‐1β. Data are presented as mean ± standard error of the mean (SEM) (*n* = 8 animals/group). Statistical significance was determined using one‐way analysis of variance (ANOVA) followed by appropriate post‐hoc testing. *p < 0.05, **p < 0.01, ***p < 0.001, ****p < 0.0001.

Exposure to SPS caused a major alteration of redox balance, with the increase in MDA and the decrease in CAT activity in animals compared to control samples (Figure 1A–C). There was a slight decrease in SOD activity, but this was not statistically significant.

One‐way ANOVA revealed a significant effect of treatment on MDA levels (*F*[5,42] = 12.54, *p* < 0.001). SPS‐exposed animals showed a marked increase in MDA compared to controls (*p* < 0.001), indicating enhanced lipid peroxidation.

Hesperidin treatment significantly reduced MDA levels across all doses, with the most pronounced reduction observed at 50 mg/kg (*p* < 0.01 vs. SPS). Higher doses (100 and 200 mg/kg) maintained this reduction without additional significant improvement. Fluoxetine also reduced MDA levels, but to a lesser extent than the hesperidin‐treated groups.

For CAT activity, a significant group effect was observed (*F*[5,42] = 09.87, *p* < 0.01). SPS exposure resulted in a significant reduction in CAT activity relative to controls (*p* < 0.01). Hesperidin treatment partially restored CAT activity, with values approaching control levels at 50 mg/kg (*p* < 0.05 vs. SPS), whereas higher doses showed no additional enhancement.

In contrast, no significant group differences were observed for SOD activity (*F*[5,42] = 2.29, *p* = 0.28), indicating relative stability of this enzymatic antioxidant across experimental conditions.

### 3.3 Hesperidin significantly attenuates SPS‐induced elevation of pro‐inflammatory cytokine

Exposure to SPS increased the levels of TNF‐α, IL‐6, and IL‐1β in the brain compared to controls, which is evidence of persistent neuroinflammatory activity (Figure 1D–F).

Hesperidin treatment significantly reduced TNF‐α levels across all doses, whereas IL‐6 showed a moderate dose‐dependent reduction. The most pronounced effect was observed for IL‐1β, where hesperidin at 50 mg/kg produced a robust decrease (*p* < 0.001 vs. SPS), exceeding the effect observed with fluoxetine. Significant group effects were observed for TNF‐α (*F*[5,42] = 14.61, *p* < 0.001), IL‐6 (*F*[5,42] = 09.46, *p* < 0.01), and IL‐1β (*F*[5,42] = 18.31, *p* < 0.001). SPS exposure significantly increased all cytokines compared to control (*p* < 0.001).

**FIGURE 2:**
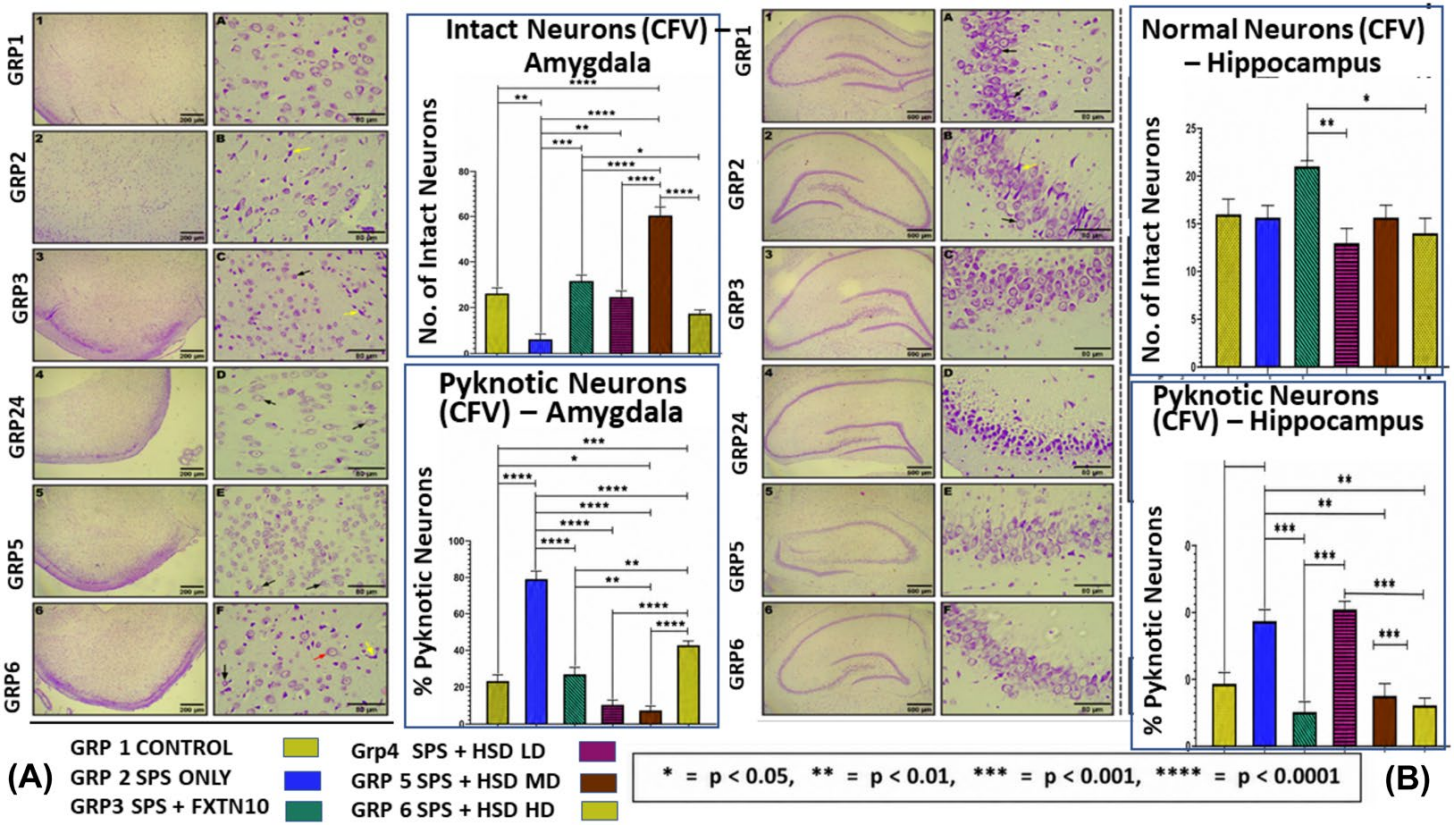
Hesperidin preserves neuronal morphology and reduces neuronal degeneration in the amygdala and hippocampus following single prolonged stress (SPS) exposure. Representative cresyl fast violet (CFV)‐stained photomicrographs and quantitative analyses of neuronal integrity in the (A) amygdala and (B) hippocampus. SPS exposure induced marked neuronal degeneration characterized by cellular shrinkage, loss of Nissl substance, and increased numbers of pyknotic neurons. Hesperidin treatment preserved neuronal architecture and significantly increased the number of morphologically intact neurons while reducing the proportion of pyknotic cells. Panel (a) shows quantitative assessment of intact neurons, whereas panel (b) shows the proportion of pyknotic neurons. Black arrows indicate normal neurons; yellow arrows indicate degenerating or pyknotic neurons. Data are presented as mean ± standard error of the mean (SEM). Scale bars: low magnification = 200–500 μm; high magnification = 80 μm. Statistical significance is indicated as *p < 0.05, **p < 0.01, ***p < 0.001, ****p < 0.0001.

**FIGURE 3:**
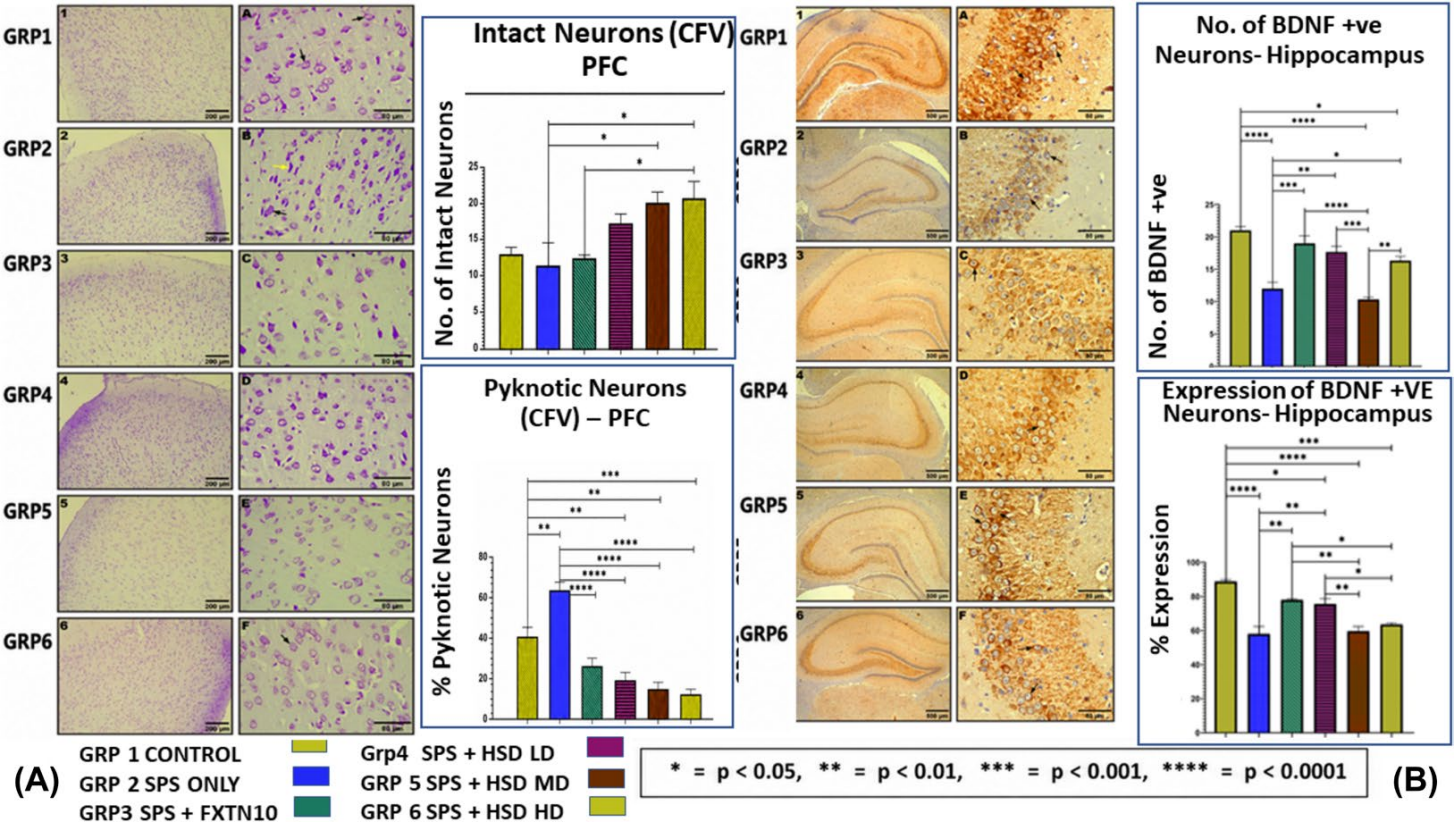
Effects of hesperidin on neuronal integrity in the prefrontal cortex and hippocampal brain‐derived neurotrophic factor (BDNF) immunoreactivity following single prolonged stress (SPS) exposure. (A) Representative cresyl fast violet‐stained sections and quantitative analyses of neuronal integrity in the prefrontal cortex. SPS exposure induced neuronal degeneration and increased pyknosis, whereas hesperidin treatment preserved neuronal morphology and increased the proportion of intact neurons. (B) Representative immunohistochemical staining for BDNF in the hippocampus. SPS markedly reduced BDNF‐positive neuronal profiles, whereas hesperidin treatment restored BDNF immunoreactivity in a dose‐dependent manner. Panel (a) shows the number of BDNF‐positive neurons, and panel (b) shows percentage BDNF expression. Black arrows indicate BDNF‐immunoreactive cells. Data are expressed as mean ± standard error of the mean (SEM). Scale bars: 80–500 μm. Statistical significance is indicated as *p < 0.05, **p < 0.01, ***p < 0.001, ****p < 0.0001.

Cresyl violet staining also showed widespread neuronal degeneration, cellular shrinkage, and pyknosis in SPS‐exposed animals in all the brain regions studied (Figures 2 and 3). The quantitative analysis showed that there was a significant decrease in morphologically intact cells and increases in the proportion of pyknotic cells compared to controls.

Photomicrographs (Figures 2 and 3) are representative images selected based on staining quality and anatomical clarity. Sections may originate from either hemisphere and slightly varying rostrocaudal levels; however, quantitative analyses were performed across multiple sections and fields and averaged per animal.

**FIGURE 4:**
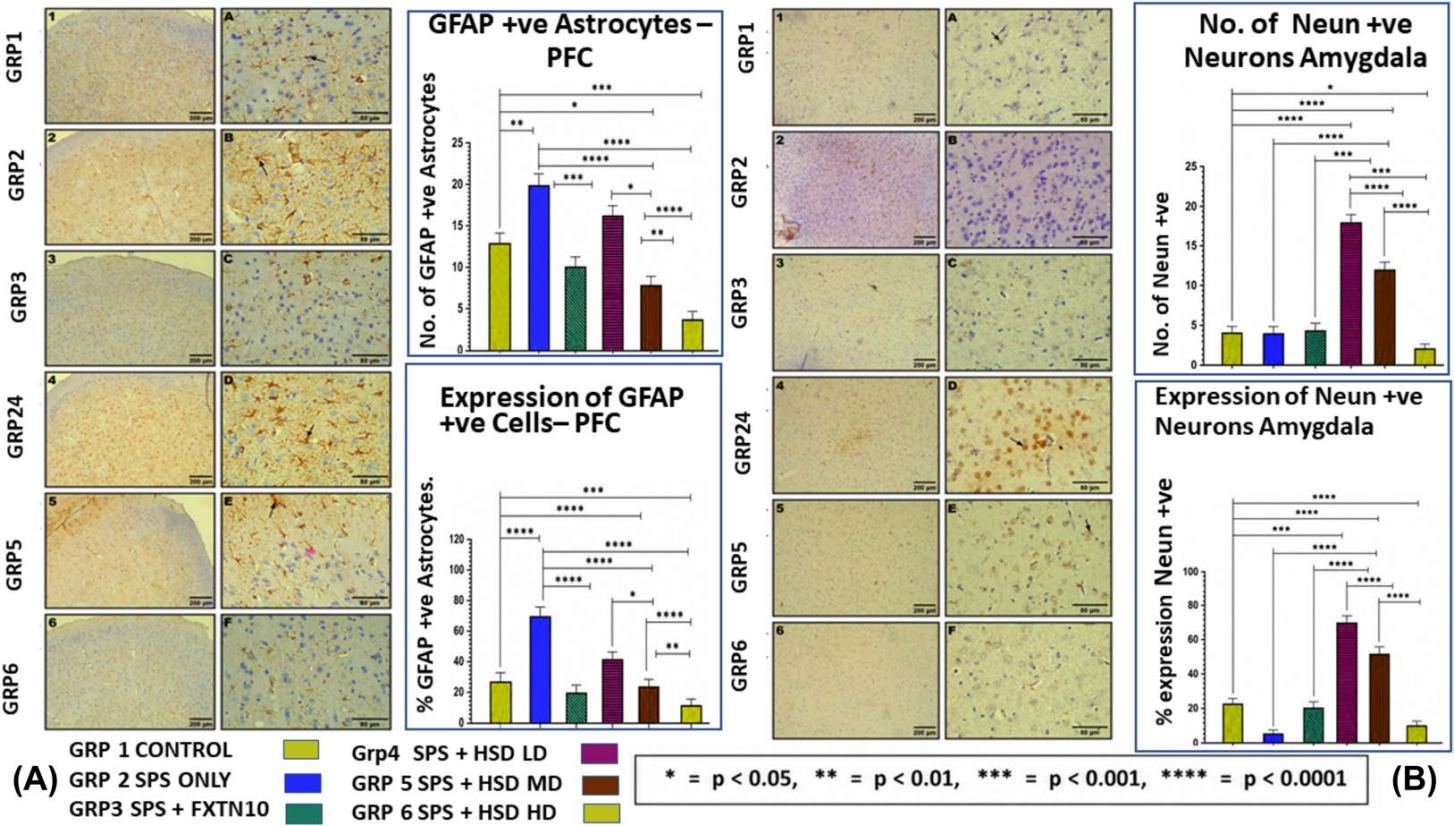
Hesperidin modulates astrocytic reactivity and preserves neuronal phenotype in the prefrontal cortex and amygdala. (A) Representative glial fibrillary acidic protein (GFAP) immunostaining and quantitative analyses of astrocytic activation in the prefrontal cortex. Single prolonged stress (SPS) exposure increased GFAP‐positive astrocytes, indicating reactive gliosis, whereas hesperidin significantly reduced astrocytic activation. (B) Representative neuronal nuclei (NeuN) immunostaining and quantitative analyses in the amygdala. SPS exposure reduced NeuN immunoreactivity, reflecting neuronal loss or phenotypic disruption, whereas hesperidin treatment restored NeuN‐positive neuronal populations. Panel (a) shows cell counts, and panel (b) shows percentage expression. Black arrows indicate positively stained cells. Data are presented as mean ± standard error of the mean (SEM). Scale bars: 80–200 μm. Statistical significance is indicated as *p < 0.05, **p < 0.01, ***p < 0.001, ****p < 0.0001.

**FIGURE 5:**
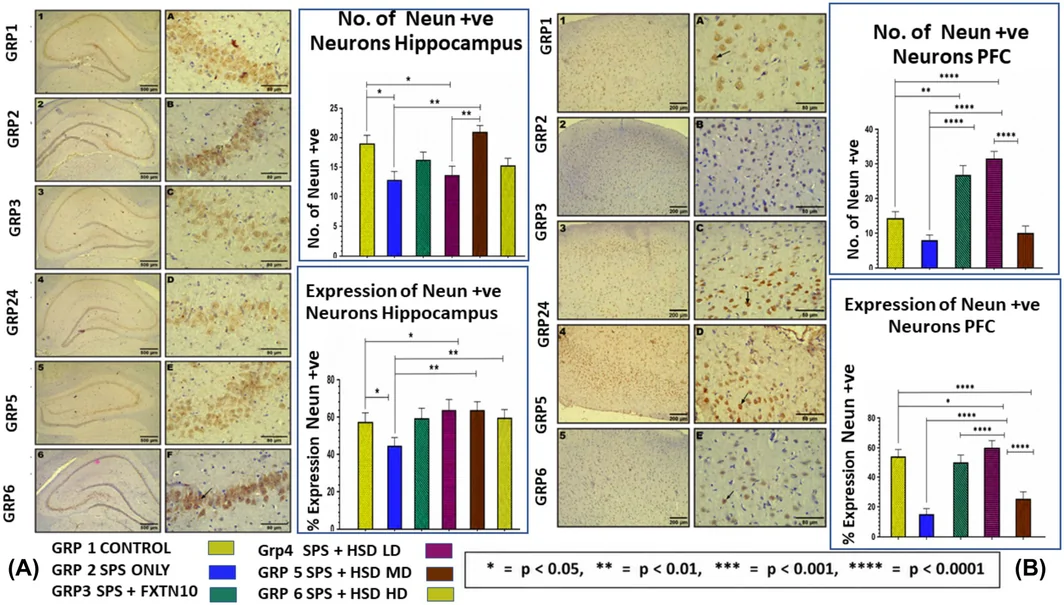
Hesperidin restores neuronal nuclei (NeuN) expression in the hippocampus and prefrontal cortex following single prolonged stress (SPS)‐induced neuronal injury. Representative NeuN immunohistochemical staining and quantitative analyses of mature neuronal populations in the (A) hippocampus and (B) prefrontal cortex. SPS exposure significantly reduced NeuN‐positive neurons and NeuN expression, indicating compromised neuronal integrity. Hesperidin treatment preserved neuronal phenotype and restored NeuN immunoreactivity in a region‐dependent manner, with greater neuroprotective effects observed at low‐to‐intermediate doses. Panel (a) shows the number of NeuN‐positive neurons, and panel (b) shows percentage NeuN expression. Black arrows indicate NeuN‐immunoreactive neurons. Data are presented as mean ± standard error of the mean (SEM). Scale bars: low magnification = 200–500 μm; high magnification = 80 μm. Statistical significance is indicated as *p < 0.05, **p < 0.01, ***p < 0.001, ****p < 0.0001. BDNF, brain‐derived neurotrophic factor; CFV, cresyl fast violet; FXT, fluoxetine (20 mg/kg); GFAP, glial fibrillary acidic protein; HSD (HD), hesperidin 200 mg/kg; HSD (LD), hesperidin 50 mg/kg; HSD (MD), hesperidin 100 mg/kg; NeuN, neuronal nuclei.

Hesperidin treatment was able to preserve neuronal morphology in a region‐ and dose‐dependent manner. Low‐to‐intermediate doses (50–100 mg/kg) really enhanced neuronal survival and minimized pyknosis. This structural observation was supported by NeuN immunohistochemistry that revealed that mature neuronal phenotype was maintained after the administration of hesperidin (Figures 4 and 5).

### 3.4 Hesperidin attenuates SPS‐induced astrocytic reactivity as evidenced by reduced GFAP immunoreactivity

**FIGURE 6:**
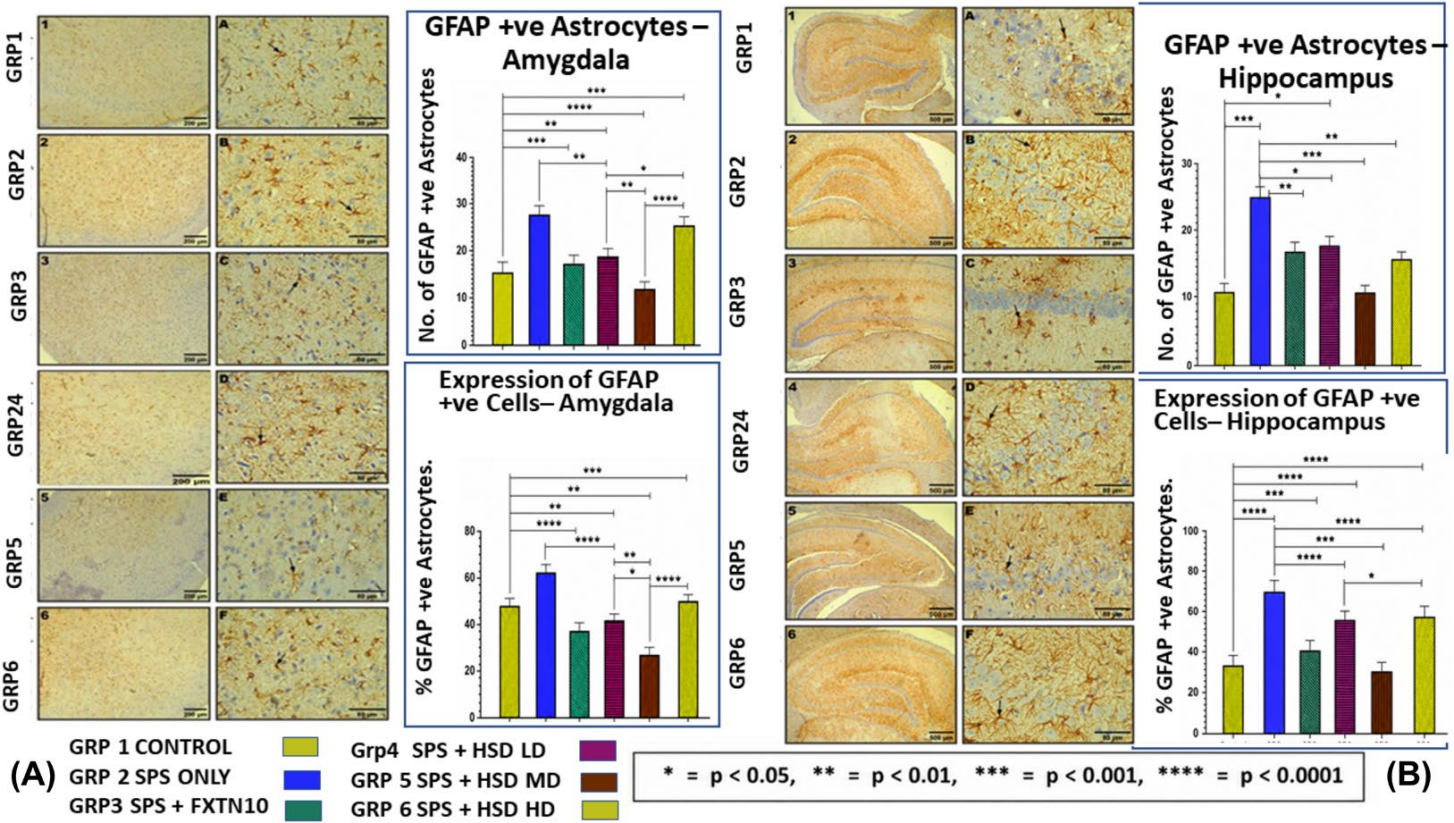
Effects of hesperidin on astrocytic activation in the amygdala and hippocampus following single prolonged stress (SPS) exposure. Representative immunohistochemical staining and quantitative analyses of glial fibrillary acidic protein (GFAP)‐positive astrocytes in the (A) amygdala and (B) hippocampus. SPS exposure induced robust astrocytic activation characterized by increased GFAP immunoreactivity and hypertrophic astrocytic morphology. Hesperidin treatment significantly attenuated reactive astrogliosis and reduced GFAP expression across both brain regions. Panel (a) shows the number of GFAP‐positive astrocytes, and panel (b) shows percentage GFAP expression. Black arrows indicate GFAP‐immunoreactive astrocytes. Data are presented as mean ± standard error of the mean (SEM). Scale bars: 80–500 μm. Statistical significance is indicated as *p < 0.05, **p < 0.01, ***p < 0.001, ****p < 0.0001.

GFAP immunostaining revealed a strong astrocytic response following SPS exposure, which is a sign of reactive gliosis (Figures 4 and 6). Treatment with hesperidin showed a significant attenuation of GFAP immunoreactivity, with the maximum reduction observed at 100 mg/kg. Interestingly, a seemingly nonlinear dose–response correlation was observed in the highest dose (200 mg/kg), which was less effective than the lower doses.

### 3.5 Hesperidin restores BDNF expression and neurotrophic support following SPS exposure

**FIGURE 7:**
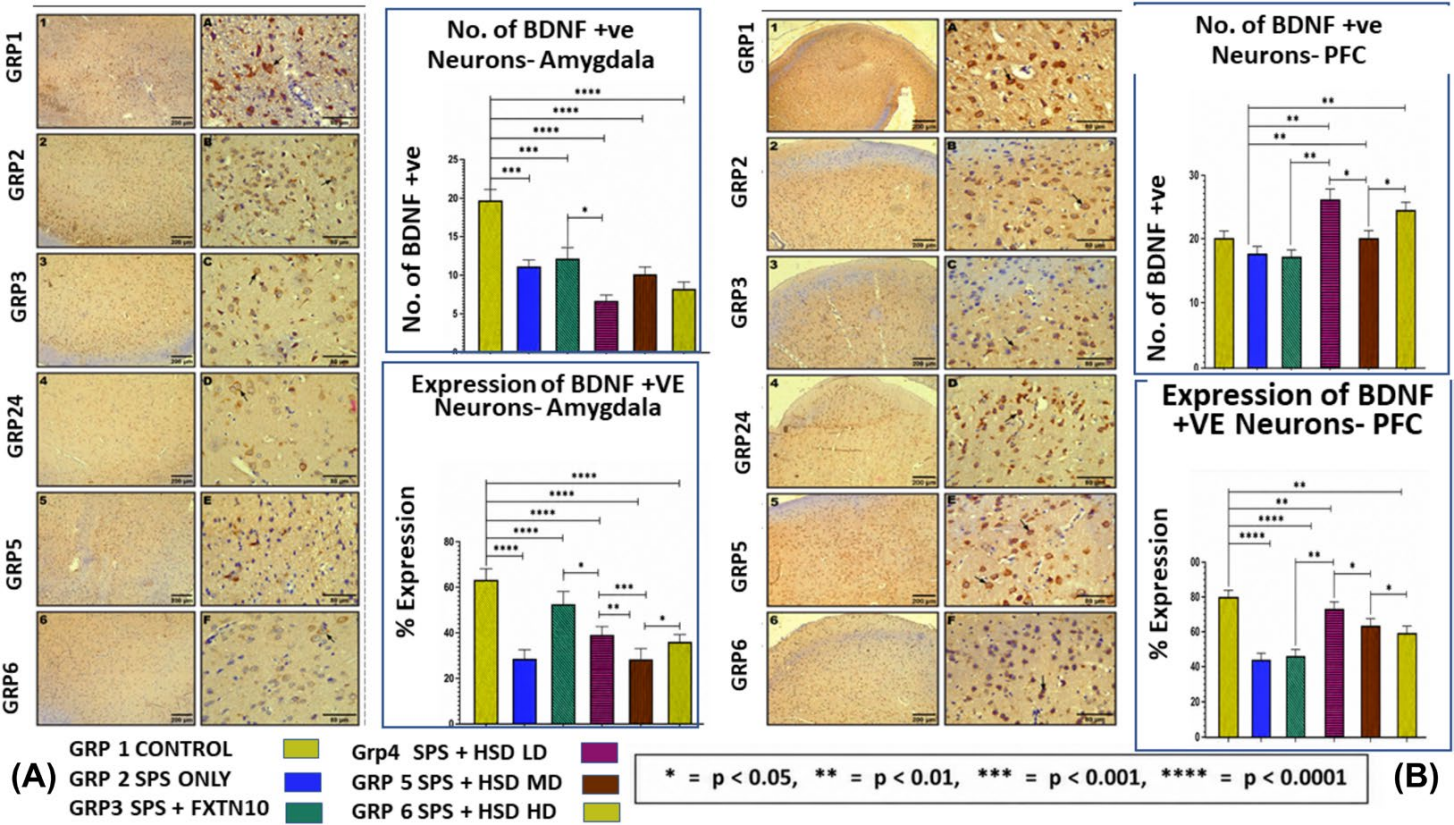
Hesperidin restores brain‐derived neurotrophic factor (BDNF) expression in the amygdala and prefrontal cortex following single prolonged stress (SPS)‐induced stress. Representative photomicrographs and quantitative analyses of BDNF immunoreactivity in the (A) amygdala and (B) prefrontal cortex. SPS exposure significantly reduced the number and expression of BDNF‐positive neurons in both regions. Hesperidin treatment restored neurotrophic signaling, with the most pronounced effects observed at low‐to‐intermediate doses. Panel (a) represents the number of BDNF‐positive neurons, whereas panel (b) represents percentage BDNF expression. Black arrows indicate positively stained neurons. Data are presented as mean ± standard error of the mean (SEM). Scale bars: low magnification = 200 μm; high magnification = 80 μm. Statistical significance is indicated as *p < 0.05, **p < 0.01, ***p < 0.001, ****p < 0.0001.

SPS exposure led to a significant decrease in immunoreactivity of the BDNF, especially in the hippocampus (Figures 3 and 7). Hesperidin administration restored the expression of the neurotrophic factor in a dose‐dependent effect, with maximal recovery observed at 100 mg/kg. Recovery within the amygdala and medial prefrontal cortex was also observed but was relatively modest.

### 3.6 Hesperidin exhibits coordinated multipathway neuroprotective effects across oxidative, inflammatory, glial, and neuronal domains

Across oxidative, inflammatory, glial, and neuronal domains, hesperidin demonstrated a coordinated modulatory profile, with optimal efficacy observed at 50–100 mg/kg. At these doses, hesperidin generated multipathway normalization equivalent in effect and, occasionally, superior to that of fluoxetine.

Taken together, these results suggest that hesperidin has a widespread neuroprotective effect in the SPS model and acts through convergent molecular and cellular mechanisms underlying stress‐induced neuropathology.

## 4 DISCUSSION

The present study shows that hesperidin modulates convergent oxidative, inflammatory, glial, and neurotrophic disturbances caused by the SPS model of PTSD. By integrating biochemical indices of redox status, cytokine profiling, and region‐specific histological and immunohistochemical analyses within a validated stress paradigm, this work advances mechanistic understanding of how multipathway dysregulation contributes to stress‐related neuropathology and how these pathways may be coordinately modulated.

Importantly, the findings extend prior behavioral validation of this model by identifying molecular and cellular correlates that plausibly underlie sustained anxiety‐like behavior and anhedonia previously reported using the same experimental framework.^30^

Building on these observations, oxidative dysregulation emerges as a central upstream mechanism driving SPS‐induced neuropathological alterations. The identified pathological features of SPS exposure include oxidative stress, which is evidenced by the high level of lipid peroxidation and the impaired antioxidant defense systems. These results are in line with mounting evidence that sustained glucocorticoid signaling and mitochondrial dysfunction induced by stress are the driving forces behind excessive production of reactive oxygen species (ROS) in PTSD.^13^, ^37^ Reduction in CAT activity observed indicates impaired hydrogen peroxide detoxification, a mechanism that enhances downstream inflammatory and apoptotic signaling.

Hesperidin selectively restored CAT activity and lowered MDA concentrations, which indicates that selective reinforcement of peroxide‐processing mechanisms, and not random scavenging of ROS, occurred. This pattern is remarkable as CAT‐dependent detoxification is of special interest in neurons as hydrogen peroxide is an important redox signaling intermediate. The lack of any meaningful impact on SOD activity also points to a specific redox‐modulatory activity as opposed to an extensive antioxidant overcorrection.^13^, ^38^

An important observation in the present study is the apparent dissociation between lipid peroxidation and antioxidant enzyme activity. Although the exposure to SPS led to a significant rise in the levels of MDA, indicating a substantial degree of membrane lipid damage, the relative changes in the levels of endogenous antioxidant enzymes, in particular, SOD, were relatively small. Such deviation is probably the consequence of inherent differences in the biology of these markers and in their kinetics.

MDA is a cumulative downstream product of oxidative damage and is very sensitive to long‐term exposure to ROS, whereas antioxidant enzymes such as SOD and CAT are tightly regulated and can exhibit adaptive/compensatory responses that are not proportional to oxidative load. Hesperidin may be acting through the selective inhibition of ROS‐induced damage and potential upregulation of oxidative systems, rather than a general increase in endogenous antioxidant defenses, which could explain the fact that hesperidin is able to substantially inhibit lipid peroxidation although it has little or no effect on SOD or a partial recovery of CAT activity. This trend contributes to the notion of pathway‐specific redox modulation during neuropathology caused by stress.

In parallel with oxidative imbalance, modulation of pro‐inflammatory cytokines represents a critical component of the neurobiological response to SPS exposure. Neuroinflammation is a pivotal link in the relationship of oxidative stress and structural neural damage in the pathogenesis of PTSD. SPS‐induced increases in TNF‐α, IL‐6, and IL‐1β found in this study are consistent with previous reports of associations between chronic stress and chronic cytokine signaling and impaired synaptic plasticity.^14^, ^39^ Among these mediators, IL‐1β is of special interest, as it is known to be involved in inhibiting hippocampal neurogenesis and long‐term potentiation.^40^, ^41^

Hesperidin caused strong inhibition of IL‐1β, which was higher than that of the antidepressant fluoxetine, but moderate and dose‐dependent inhibition of TNF‐α and IL‐6. This selectivity of cytokine is in line with previous data reporting that hesperidin can mediate nuclear factor kappa‐light‐chain‐enhancer of activated B cells (NF‐κB) and NLRP3 signaling.^26^ By preferentially attenuating IL‐1β, hesperidin may disrupt self‐sustaining inflammatory feedback loops that perpetuate neuronal vulnerability following stress exposure.

In addition to neuronal and inflammatory changes, astrocytic reactivity emerges as a key intermediate feature of SPS‐induced neuropathology. Reactive astrogliosis is emerging as an established maladaptive factor of chronic stress pathology, with impacts on the synaptic homeostasis, glutamate clearance, and neuroinflammatory amplification.^27^, ^42^, ^43^ In the current work, the astrocytic activation in stress‐sensitive areas was confirmed by SPS increases in GFAP expression levels.

Hesperidin decreased GFAP immunoreactivity and did not fully suppress astrocyte activity, implying maladaptive reactivity inhibition without the elimination of supportive glial activities. The observed nonlinear dose–response, in which the largest dose had less efficacy, also suggests the notion that the optimal modulation (as opposed to maximal suppression) of glial activation may be neuroprotective. This trend is consistent with new opinions that too much glial inhibition can impair metabolic and trophic maintenance of neurons.^44^, ^45^, ^46^, ^47^

Consistent with these upstream effects, preservation of neuronal structure and phenotypic integrity represents a key outcome of the observed neuroprotective processes. Histology showed that neuronal degeneration and pyknosis occurred extensively after exposure to SPS, which is also observed in the hippocampus, amygdala, and prefrontal cortex in response to stress. The hesperidin‐treated group exhibited neuronal preservation and maintenance of phenotypic integrity. Preservation of NeuN immunoreactivity and normal neuronal morphology after hesperidin treatment is widely interpreted as evidence of true neuroprotection and maintenance of mature neurons, not just a slowing of cell death.^48^, ^49^

These findings indicate that hesperidin's effects extend beyond biochemical normalization to structural preservation, potentially by reducing cumulative oxidative and inflammatory insults that destabilize neuronal identity. The alignment between Nissl staining (cell body integrity) and NeuN expression (mature neuronal marker) supports the interpretation that hesperidin truly preserves neuronal integrity rather than cosmetically masking injury.

Reduced BDNF expression is a consistent feature of neuropathology of PTSD and a crucial determinant of synaptic plasticity and stress resilience.^50^, ^51^ The observed significant decrease in hippocampal BDNF following SPS exposure in this study is consistent with this framework. Hesperidin restored immunoreactivity of BDNF in a dose‐dependent manner, with peak recovery at intermediate doses. This restoration is probably the indirect result of normalization of the redox‐inflammatory environment rather than direct trophic stimulation that is required to provide a permissive environment for synaptic maintenance and remodeling. These molecular findings augment earlier‐reported behavioral evidence of enhanced anxiety‐ and depression‐related outcomes within the same treatment framework.^30^

The data collectively endorse a concept wherein SPS triggers oxidative dysregulation, which enhances neuroinflammatory signaling, fosters maladaptive astrocytic activation, and undermines neuronal integrity and trophic support. Hesperidin seems to act at several nodes of this network, and the most effective action was found to be at the intermediate doses (50–100 mg/kg). The overlapping of redox, immune, glial, and neuronal modulation implies the association of pathway control, as opposed to independent target action.

Fluoxetine was used as a mechanistic comparator, which suggests pathway selectivity instead of therapeutic advantage. Although fluoxetine partially restored inflammatory and oxidative markers, hesperidin showed a wider modulation effect, both structurally and trophically.

There are a number of limitations that should be considered. Only male animals were used; therefore, sex‐specific effects could not be assessed. Microglial phenotyping, synaptic ultrastructure, and functional connectivity were also not assessed. A further limitation is the absence of direct endocrine assessment of HPA axis activation (e.g., serum corticosterone levels), which, although well established in the SPS model, would provide an additional layer of physiological validation.

Future studies incorporating longitudinal behavioral–molecular coupling, microglial markers, synaptic protein analysis, and female cohorts will be essential to refine mechanistic specificity and translational relevance.

In conclusion, the present study does not define hesperidin as a potential therapeutic agent in the management of PTSD but as a mechanistic aid in the investigations of the integrated redox‐immune‐glia‐trophic control in experimental stress models. The findings are important to the deeper comprehension of stress‐induced neuropathology, as they demonstrate the coordinated, dose‐sensitive modulation in various pathological domains and provide important nodes to be used in future intervention measures.

## AUTHOR CONTRIBUTIONS

**Godson Emeka Anyanwu:** Conceptualization; project administration; supervision; validation; visualization; writing – original draft; writing – review and editing. **Ogan Christopher Akanaku:** Conceptualization; formal analysis; funding acquisition; investigation; methodology; writing – original draft; writing – review and editing. **Augustine Oviosun:** Data curation; investigation; methodology; validation; visualization; writing – original draft; writing – review and editing. **Precious E. Ekwueme:** Formal analysis; software; visualization; writing – original draft; writing – review and editing. **Paul Anyiom Odey:** Investigation; methodology; project administration; writing – original draft. **Chima Paul Okechukwu Ugwu:** Supervision; validation; visualization; writing – original draft; writing – review and editing. **Chinyere Nkemjika Anyanwu:** Data curation; project administration; supervision; validation; visualization; writing – original draft; writing – review and editing.

## FUNDING INFORMATION

This research received no external funding.

## CONFLICT OF INTEREST STATEMENT

The authors declare no conflicts of interest.

## ETHICS STATEMENT

All animal procedures were conducted in accordance with the National Institutes of Health (NIH) Guide for the Care and Use of Laboratory Animals and approved by the Research Ethics Committee of the College of Medicine, University of Nigeria, Enugu Campus (approval number: COMHREC/2025/01/002).

## Data Availability

Data will be made available on reasonable request.
